# Habit-like attentional bias is unlike goal-driven attentional bias against spatial updating

**DOI:** 10.1186/s41235-022-00404-7

**Published:** 2022-06-17

**Authors:** Injae Hong, Min-Shik Kim

**Affiliations:** Department of Psychology, Yonsei University, Yonsei-ro 50 Seodaemun-gu, Seoul, 03722 Korea

**Keywords:** Habit-like attention, Goal-driven attention, Location probability learning, Attentional bias, Visual search

## Abstract

Statistical knowledge of a target’s location may benefit visual search, and rapidly understanding the changes in regularity would increase the adaptability in visual search situations where fast and accurate performance is required. The current study tested the sources of statistical knowledge—explicitly-given instruction or experience-driven learning—and whether they affect the speed and location spatial attention is guided. Participants performed a visual search task with a statistical regularity to bias one quadrant (“old-rich” condition) in the training phase, followed by another quadrant (“new-rich” condition) in the switching phase. The “instruction” group was explicitly instructed on the regularity, whereas the “no-instruction” group was not. It was expected that the instruction group would rely on goal-driven attention (using regularities with explicit top-down knowledge), and the no-instruction group would rely on habit-like attention (learning regularities through repetitive experiences) in visual search. Compared with the no-instruction group, the instruction group readjusted spatial attention following the regularity switch more rapidly. The instruction group showed greater attentional bias toward the new-rich quadrant than the old-rich quadrant; however, the no-instruction group showed a similar extent of attentional bias to two rich quadrants. The current study suggests that the source of statistical knowledge can affect attentional allocation. Moreover, habit-like attention, a different type of attentional source than goal-driven attention, is relatively implicit and inflexible.

**Supplementary Information:**

The online version contains supplementary material available at 10.1186/s41235-022-00404-7.

## Significance statement

Visual search is ubiquitous in our lives, and knowing statistical knowledge about the target’s location would facilitate visual search. Implicit learning of the target’s location probability causes the habitual attentional shift, suggesting the visual system’s adaptability to complex visual scenes. However, such an adaptive system can turn into a maladaptive behavior in some situations of a rapidly changing society. For example, a habitual and persistent attentional shift would no longer be a helpful strategy when we cross over the multiple web pages in a short period. Then, how can we form an adaptive visual search strategy for the rapidly changing environment? How can we help searchers quickly adapt to a changing environment? This study stemmed from a fundamental question on the adaptability of the visual system to the dynamic search environments. The current study possesses practical implications of LPL in various fields. Explicit or implicit training should be used appropriately in critical situations. For example, the habitual LPL paradigm can be used to develop novel rehabilitation or educational programs to complement attentional orienting in stable situations, like elder people locating the necessities in their familiar space. In other cases where a search relies on a rapid readjustment of spatial bias, like online surfing or searching for a criminal, explicit knowledge that guides spatial attention would be helpful. LPL is a theoretically convenient paradigm to show the visual system’s efficiency in integrating multiple instances into a single knowledge. However, in real-life situations, more complex visual scenes should be considered.

## Introduction

Visual search, finding a target among distractors, is ubiquitous in our daily lives (e.g., finding a car in a parking lot) and in professional fields, like medical imaging (Drew et al., 2013; Sheridan & Reingold, 2017), airport scanner (Mitroff & Biggs, 2014) and sports (Piras et al., 2014). Sometimes, spatiotemporal statistical regularity lies in visual search circumstances, and repetitive search experiences result in the learning of regularity (Chun & Jiang, 1998). Location probability learning (LPL) is one of the statistical learning paradigms that facilitates visual search by spatial bias toward a target’s frequent location (Geng & Behrmann, 2002, 2005; Jiang et al., 2013b). In a typical LPL paradigm, a target frequently appears at a particular search region. Multiple search experiences with such target-related statistical regularity automatically guide spatial attention to a frequent target region, resulting in faster target detections. A long-term influence of automatic attentional guidance by LPL (Jiang et al., 2013b) is effective regardless of the developmental trajectory (Jiang et al., 2016; Lee et al., 2020; Sisk et al., 2018), search space scale (Sisk et al., 2021; Smith et al., 2010; Won et al., 2015), cognitive impairment with working memory load (Won & Jiang, 2015) or cognitive deficits (Sisk et al., 2018). In this regard, LPL paradigm is prominent as an educational or rehabilitation program to induce adaptive cognitive bias for those who have difficulty understanding explicit rules within their daily lives.

However, in real-life search situations, habitual attentional shifts by statistical learning would not always an adaptive strategy. Statistical regularities in our real-life situations are not always stable, and it can be dynamic especially in a rapidly changing society. For example, a soccer player can alter the game strategy to shoot the ball at an unexpected location, or the expected location of a tumor can vary by the patient’s initial health state. It is also common in our daily lives that one may inevitably park a car in front of a building other than the one they usually park at. Imagine that you are searching for an article and the websites you are located at are changing every 3 s. Changed statistical regularity can be gradually perceived by repetitive exposure to the regularity or by a sudden and explicit notification of the change. This leads to the question: How can we find adaptive search strategy that fits the dynamic visual searching situations? Does the knowledge source of the altered regularity, by repetitive experiences or explicit knowledge, affect the updating of spatial bias?

The question about the knowledge source of statistical regularity should be carefully examined because it concerns attentional resources used in visual search. Visual search with explicit and top-down knowledge is expected to rely on goal-driven attention (Folk et al., 1992), and learning of a target’s location probability through repetitive experiences is expected to rely on habit-like attention (Jiang, 2018).

Habit-like attention is another term for experience-driven attention and is known as the leading attentional control resource of LPL with its three principal characteristics (Jiang & Sisk, 2019; Jiang et al., 2018). Like a habit, habitual bias is gradually acquired by experience accumulation, is irrelevant to explicit prior knowledge of the rule or conscious intention to learn the rule, and is insensitive to outcome devaluation.

These characteristics are distinct from goal-driven attention (Addleman et al., 2018), where explicit knowledge flexibly shifts spatial attention. Attentional bias caused by goal-driven attention was transferrable to irrelevant memory tasks, while bias caused by implicit habit-like attention was not (Addleman et al., 2018). LPL was persistent with the explicit instruction of regularity changes (Jiang et al., 2014). Moreover, LPL is possible for those with cognitive impairment (Geng & Behrmann, 2002; Shaqiri & Anderson, 2012, 2013; Sisk et al., 2018) or aged or younger groups (Jiang et al., 2016; Lee et al., 2020), whose goal-driven attentional control ability is relatively degraded than healthy adults. A meta-analysis aggregating the existing ~ 400 datasets (Jiang et al., 2018) showed that the recognition accuracy of a target’s frequent location, or the awareness degree of the regularity, did not predict the extent of attentional bias.

While LPL is relatively implicit and inflexible compared to attentional allocation by goal-driven attention, recent studies have raised the issue that LPL might be neither implicit nor inflexible (Giménez-Fernández et al., 2020, 2021; Vadillo et al., 2020). Giménez-Fernández et al. replicated the LPL studies using tenfold more participants (*N* = 160 vs. *N* = 16 from the existing studies’ average) as well as novel awareness measurements such as a ranking task (asking participants to rank the quadrants according to how frequently the target is expected to have appeared in them) and a probability estimation task (asking participants to assign each quadrant a percentage probability indicating the expected likelihood that the target will appear in it). As a result, the learning effect showed a diminishing trend when the target probability no longer existed (rebutting inflexibility of LPL). Also, the ranking score and the probability estimate exceeded the chance level (rebutting implicitness of LPL). Based on these results, the research group contended that the three characteristics of habit-like attention should be re-examined.

The controversy over inflexibility and implicitness should not be disregarded because these two characteristics dissociate habit-like attention from goal-driven attention (Jiang, 2018). If the claims by Giménez-Fernández et al. (2020) and Vadillo et al. (2020) are more reliable, then the habit-like attention model should be re-examined.

This study directly addresses the issue of habit-like attention versus goal-driven attention on LPL by comparing attentional bias in the face of spatial regularity updating. With a sample size of adequate statistical power, the implicitness and inflexibility of LPL were tested. The types of attentional control resources participants rely on were manipulated by the presence of explicit instructions on statistical regularity. Participants with explicit instruction were expected to rely on goal-driven attention, while participants without explicit instruction were expected to rely on habit-like attention during visual search. If goal-driven and habit-like attention are dissociable, explicitly instructed participants would rapidly and flexibly switch their attentional bias, while uninstructed participants would do so slowly and inflexibly. If they are not dissociable and habit-like attention shares common characteristics with goal-driven attention, the speed and location of spatial updating of attentional bias would be comparable. Additionally, this study answers the importance of knowledge sources on understanding statistical regularity in applied situations.

## Method

### Participants

A total of 60 participants (*N*_male_ = 5, *N*_female_ = 55; *M*_age_ = 23.7 years, *SD* = 3.11 years) were recruited online in exchange for monetary rewards. A proper sample size with an expected power of 0.95 and *α* error probability of 0.05 was 14. This was based on a previous study that tested attentional bias by explicit knowledge (Jiang et al., 2014) and obtained *η*_p_^2^ = 0.53 with 12 samples. We finalized 16 valid datasets for two independent groups (a total of 32 datasets) following the dropping criteria (see “Analysis” section).

### Apparatus, stimuli, and procedure

The experiment was programmed with PsychoPy (Peirce et al., 2019) and ran online on the Pavlovia.org server. Eight blocks were included in the training phase, and two blocks in the switching phase. Each phase contained 48 trials. A practice phase using 48 trials was conducted before the main experimental session.

**Fig. 1:**
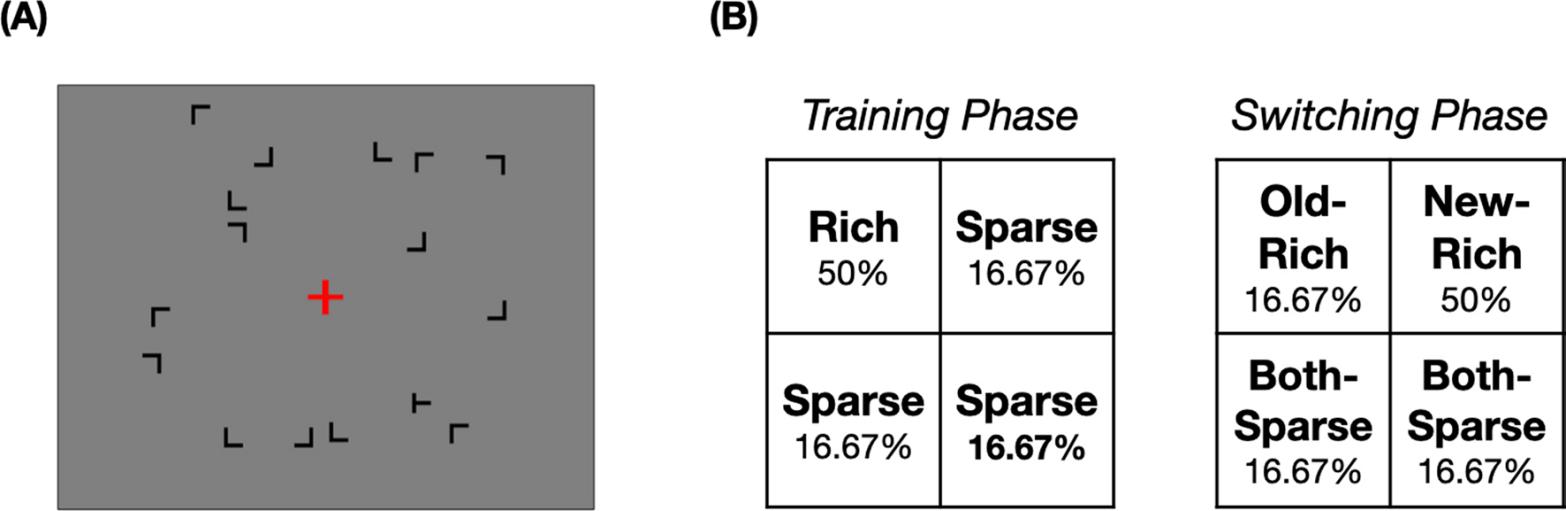
Schematic description of the experiment. **A** A sample search display. The items are not drawn to actual scale. **B** Schematic description of target probability and condition names of the training phase (left) and the switching phase (right). The rich quadrants were counterbalanced by participants

After a red fixation (20 px × 20 px; Item sizes were reported in pixels, not as visual degrees, because the experimental condition was not finely controlled due to it being an online experiment.) was presented alone for 500 ms, a search array (600 px × 600 px) with one T-shaped target (rotated 90° either to the left or to the right) and 15 L-shaped distractors (rotated 0°, 90°, 180°, or 270°) appeared until the participants made a response (item size: 30 px × 30px). The search item’s location was randomly selected from 10 × 10 invisible matrices, and their specific locations were randomly jittered ± 10 px horizontally and vertically from the center. The task was to press “z” for the left-tilted and “/” for the right-tilted T as fast and accurately as possible. A 1000 ms visual feedback was provided for the incorrect response. The subsequent trial began after a 500 ms interval. The sample search display is depicted in Fig. 1A.

In the training phase, the target appeared in the “rich” quadrant for 50% of the trials and in each of the “sparse” quadrants for 16.67% of the trials. In the switching phase, the rich quadrant shifted to one of the sparse quadrants, and the previously rich quadrant became a sparse quadrant. The rich quadrant during the switching phase was the “new-rich” quadrant, and the previously rich quadrant was the “old-rich” quadrant. The remaining two sparse quadrants were the “both-sparse” quadrants (Fig. 1B). The rich quadrants of the training and switching phases never overlapped. The two rich quadrants were counterbalanced by the participants.

Participants in the “no-instruction” group were not informed of the target probability distribution. In contrast, participants in the “instruction” group were told that the target could appear more frequently in the *first (upper-right)* quadrant (The possible instructions were the* first [upper-right], second [upper-left], third [bottom-left], or fourth [bottom-right]* quadrant.) This explicit instruction was provided once before the training phase and once before the switching phase.

After the search task, all participants completed a questionnaire sheet through GoogleDocs. First, participants in the no-instruction group were asked whether they had noticed any rule in the experiment (*noticed* or *not noticed*). If they noticed one, they were asked to write it down freely. Second, participants were asked whether they noticed that the target was frequently presented at a particular quadrant in the training and switching phases, respectively (*noticed* or *not noticed*). Third, participants were forced to choose rich quadrants for the training and switching phases. Participants in the instruction group were only given the questionnaire in the third step to confirm if they were fully aware of the rich quadrants. Five participants turned in the survey sheet twice with different answers. In this case, the second answer was considered.

### Analysis

Datasets were replaced until the number of valid datasets reached 32, based on the awareness questionnaire’s answers. The purpose of continuous replacement was to compare attentional learning between the no-instruction and instruction groups with a more conservative criterion.

Participants who correctly picked either the old-rich *or* the new-rich *or* both quadrants for the no-instruction group were excluded and substituted. Only the four-alternative forced choice (4AFC) task’s responses were considered to apply a more conservative criterion on participants’ awareness. If participants randomly guessed the rich quadrants, the number of correctly choosing at least one rich quadrant in the two 4AFC tasks would be 7 among 16 possible combinations (4 possible responses for each 4AFC task). A total of 31 participants were recruited for the no-instruction group until we finalized the datasets with 16 participants, excluding 17 datasets.

Only participants who correctly reported both the training *and* switching phase for the instruction group were selected. A total of 9 participants who failed to report two rich quadrants were replaced until the number of valid datasets reached 16. Table 1 shows the frequency of participants by the accuracy of the 4AFC task.

**Table 1:** The number of participants by 4AFC task accuracies of the training and the testing phases

Training phase	Switching phase	Frequency
*Instruction group (explicit knowledge; N* = *27)*		
Incorrect	Incorrect	0
Incorrect	Correct	3
Correct	Incorrect	8
Correct	Correct	16*
*No-Instruction group (implicit knowledge; N* = *33)*		
Incorrect	Incorrect	16*
Incorrect	Correct	5
Correct	Incorrect	7
Correct	Correct	5

The final datasets of the instruction and no-instruction groups are marked with asterisks

**Fig. 2:**
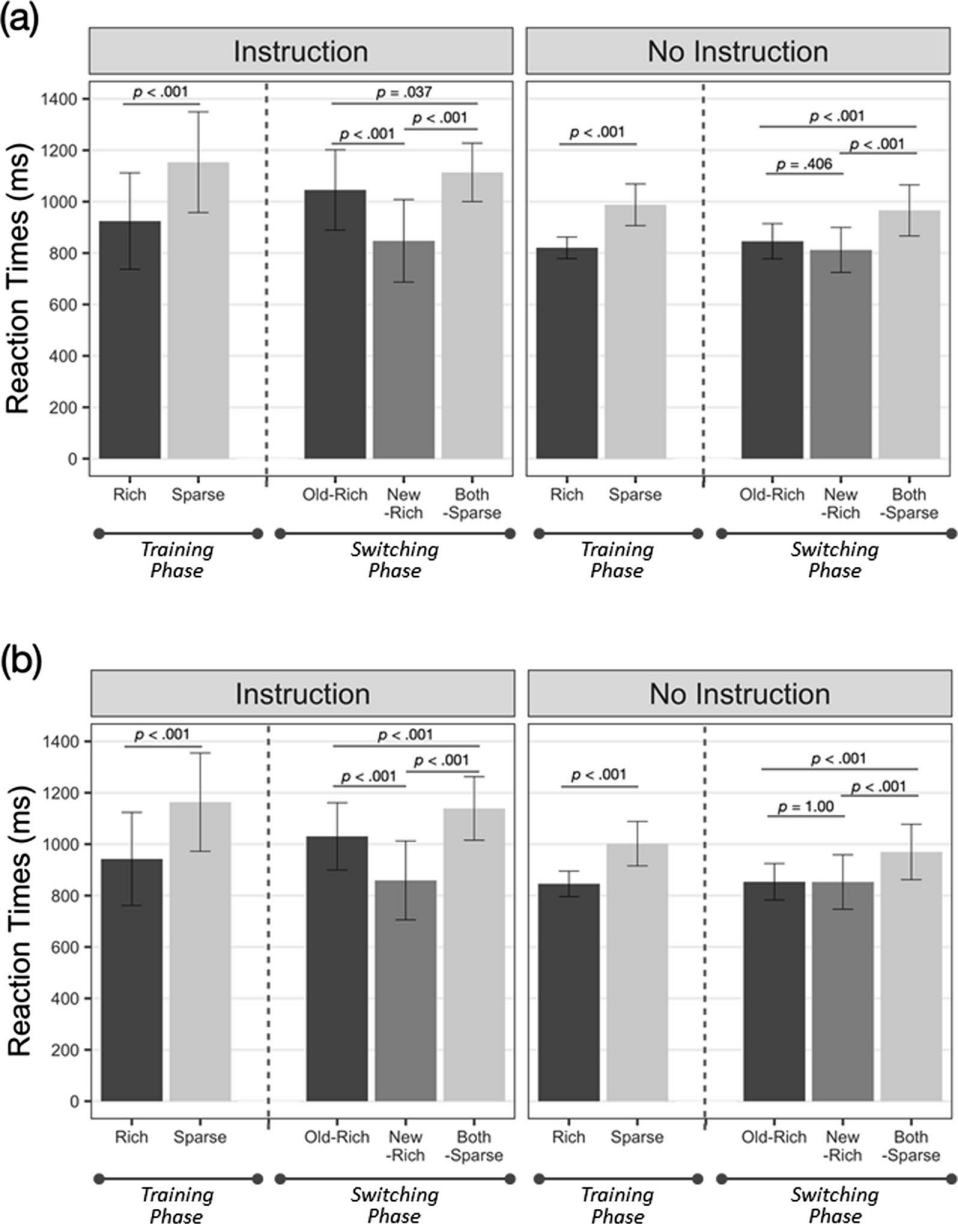
Mean RTs of instruction group and no-instruction group. *Note*: **a** RTs of datasets whose awareness score was reflected (*N* = 32). **b** RTs of the complete datasets whose awareness score was not reflected (*N* = 60). Error bars represent 95% confidence intervals. The descriptive statistics of RTs can be found in Additional file 1: Table S1.

Correct RTs between 200 ms and 10 s were analyzed using a generalized linear mixed-effect model (GLMM) with the *afex* package (Baayen et al., 2008). Independent GLMM tests were conducted for the training and switching phases. The fixed effects of the training phase were the target’s location (rich, sparse) and the awareness state (instruction, no-instruction). The fixed effects of the switching phase were the target’s location with three levels (old-rich, new-rich, both-sparse) and the awareness state. By-subject intercepts were included as a random effect. RTs were fitted to the inverse Gamma function. Significance tests against the null model were conducted with the likelihood ratio test (Singmann et al., 2020). The *p* values of the post-hoc analysis were adjusted using a Bonferroni method. The mean RTs are plotted in Fig. 2.

## Results

### Spatial updating with experience-driven and goal-driven knowledge

RTs of 32 datasets (16 sets from each group) among 60 were analyzed to examine whether the attentional bias by LPL is affected by explicit goal-driven or implicit experience-driven knowledge of the target distribution. A total of 0.039% trials were excluded based on the RTs exclusion criterion. Overall accuracy was 98.71% (*SD* = 1.28%) for the instruction group and 96.62% (*SD* = 3.50%) for the no-instruction group, *t*(30) =  − 0.240, *p* = 0.032. RTs were mainly analyzed as a dependent variable of interest, but additional analysis addressed possible RT-accuracy trade-off in Additional file 1.

First, the RTs of the training phase were analyzed. The fixed effect of the target’s location was significant, *χ*^*2*^(1) = 938.700, *p* < 0.001, and the fixed effect of the awareness state was not significant, *χ*^*2*^(1) = 1.641, *p* = 0.200. The target’s location × awareness state interaction was not significant, *χ*^*2*^(1) = 0.238, *p* = 0.625. Both the instruction and no-instruction groups showed attentional bias toward the rich quadrant than the sparse quadrants, instruction group *z* = 23.49, *p* < 0.001; no-instruction group *z* = 20.62, *p* < 0.001. The results from the training phase indicate that attentional bias toward the rich quadrant emerged independently of the learning source.

Did the awareness states modulate spatial updating? The regularity was suddenly changed to bias different quadrant in the switching phase, and this fact was either notified (instruction group) or not notified (no-instruction group) to the participants. To test whether the attentional bias was updated or not by a novel regularity, RTs of the switching phase were analyzed. The fixed effect of the target’s location was significant, *χ*^*2*^(2) = 230.505, *p* < 0.001. The interaction between the awareness state and the target’s location was significant, *χ*^*2*^(2) = 19.606, *p* < 0.001. For the instruction group, RTs of the new-rich condition were faster than both the old-rich, *z* =  − 8.411, *p* < 0.001, and both-sparse conditions, *z* = 10.893, *p* < 0.001. RTs of the old-rich and both-sparse conditions were significantly different, *z* = 2.500, *p* = 0.037. For the no-instruction group, both the old-rich and new-rich conditions showed significantly faster RTs than the both-sparse conditions, old-rich *z* = 4.784, *p* < 0.001, new-rich *z* = 8.552, *p* < 0.001. RTs of the old-rich and new-rich conditions were not significantly different, *z* =  − 1.494, *p* = 0.406. RTs by awareness state were not significantly different, *χ*^*2*^(1) = 2.270, *p* = 0.132. While those with explicit knowledge on novel regularity showed the most attentional priority to the novel rich quadrant, those with implicit knowledge showed persistent attentional bias toward both, the old- and new-rich quadrants.

Because the target was more likely to be presented in the new-rich quadrant in the switching phase, a short-term repetition priming (Brascamp et al., 2011; Maljkovic & Nakayama, 1996) could have facilitated visual search, not LPL. Therefore, additional analysis was conducted on the switching phase by removing the trials whose target quadrant was repeated from the previous trial. The additional analysis showed a similar trend of results (see the Additional file 1 and Figure S1).

In sum, attentional bias was more considerable when participants were trained implicitly than explicitly to bias one rich quadrant. When a novel regularity was introduced, participants used different search strategies. Participants with explicit knowledge of the novel regularity rapidly updated attentional priority. The implicitly trained participants showed persistent attentional bias toward the previously rich quadrant and comparable attentional bias toward the recent rich quadrant.

### Spatial updating regardless of awareness level

Due to a conservative criterion on the explicitness and implicitness of LPL, 28 out of 60 datasets were dropped out. The high dropout rate could have affected the statistical test; thus, an additional analysis with complete datasets was conducted. The exclusion criterion of RTs removed 0.13%. The mean accuracy of the instruction group was 98.65% (*SD* = 1.18%), and that of the no-instruction group was 96.87% (*SD* = 3.19%), *t*(58) =  − 2.842, *p* = 0.008 (see Additional file 1 for RT-accuracy trade-off analysis).

In the training phase, the fixed effect of the target’s location was significant, *χ*^*2*^(1) = 2479.897, *p* < 0.001. The target’s location × awareness state interaction was not significant, *χ*^*2*^(1) = 0.785, *p* = 0.378; instruction group *z* = 36.63, *p* < 0.001; no-instruction group *z* = 34.78, *p* < 0.001. The fixed effect of the awareness state was not significant, *χ*^*2*^(1) = 2.134, *p* = 0.128.

In the switching phase, the fixed effect of the target’s location was significant, *χ*^*2*^(2) = 338.687, *p* < 0.001, and the interaction between the target’s location and awareness state was significant, *χ*^*2*^(2) = 26.957, *p* < 0.001. RTs by awareness state did not show statistical difference, *χ*^*2*^(1) = 2.085, *p* = 0.149. In specific, participants with explicit instruction showed the fastest RTs in the new-rich condition than the old-rich condition, *z* =  − 7.546, *p* < 0.001, and the both-sparse conditions, *z* = 15.613, *p* < 0.001. RTs of the old-rich condition were faster than the both-sparse conditions, *z* = 4.545, *p* < 0.001. Participants with implicit knowledge showed more attentional priority to both the old-rich, *z* = 7.336, *p* < 0.001, and new-rich quadrants, *z* = 10.886, *p* < 0.001, than the both-sparse quadrants. However, the RTs of the old-rich and the new-rich quadrants were not statistically significant, *z* =  − 0.527, *p* > 0.99.

Overall, including all the datasets regardless of awareness level yielded similar results to the subsets of data by awareness level. Those with explicit knowledge rapidly updated attentional priority despite regularity change. In contrast, those with implicit knowledge showed comparable attentional bias toward old-rich and new-rich quadrants.

## Discussion

The current study tested the availability of spatial updating following regularity change with different attentional resources. Explicit knowledge of target probability was provided (instruction group) or not (no-instruction), to manipulate the type of attentional resources participants rely on for visual search. Explicit knowledge of novel regularity enabled a quicker shift of attentional priority from the previously frequent target location to the novel frequent target location. In contrast, implicit learning of novel regularity allowed multiple prioritizations of possible target locations by maintaining previous implicit knowledge and learning a novel regularity. While spatial updating was relatively fast and flexible with explicit goal-driven attention, it was relatively slow and inflexible with implicit habit-like attention.

We precisely censored participants according to their awareness of the search regularity with conservative criteria. One potential concern is that around half of the total participants were eliminated. Nevertheless, the biasing pattern of the selected participants was not qualitatively different from that of the entire sample. The conservative censoring criteria enabled a direct comparison of attentional biasing with explicit or implicit knowledge of location probability.

The visual search task and the relevant statistical regularity were the same for the instruction and no-instruction groups except for the explicitness of the regularity. Yet, the no-instruction group generally showed faster target detection performance but lower accuracy (see “Results” section). The reasons for this difference are not known but may be inferred. The two groups could have relied on different cognitive resources, or at least different search strategies, derived from different instructions. By the no-instruction group relying on habit-like attention, implicit procedural memory of the target’s frequent location primarily guided spatial attention (Jiang et al., 2013a). On the other hand, the instruction group relied on goal-driven attention in visual search by utilizing explicit knowledge of the target’s location. By the instruction group verbalized the procedural memory, impairing motor performance (Chauvel et al., 2013).

Nevertheless, the difference in RTs by awareness state is not worrisome. First, the extent of attentional bias by LPL is irrespective of different RT levels (caused by different task difficulties; Jiang et al., 2015). Second, we observed rapid (and slow) readjustment of attentional bias in the instruction (and no-instruction) group when RT-accuracy trade-off was considered (see Additional file 1). Likewise, the different extent of attentional bias by awareness state levels cannot be attributed to general search ability.

The different biasing pattern as well as different RTs seem to reflect different cognitive processes under instruction and no-instruction groups. The statistical regularity on the target’s location was exactly identical for both the instruction and no-instruction groups, but the nature of statistical knowledge they are relying on changed the attentional prioritization strategy. While statistical knowledge of the instruction group is semantic and verbalized, that of the no-instruction group is procedural and non-verbalized. In the case of the instruction group, the instruction of the switching phase conflicts with that of the learning phase, so the latest knowledge that fits the current regularity becomes the winning knowledge. Visual search of the instruction group is not generally affected by the accumulated information about the target’s location (Horowitz & Wolfe, 1998) and is refreshed every search episode. However, in the case of the no-instruction group, procedural knowledge is accumulated in an experience-driven manner. Therefore, it takes time to verify that biasing the previously-rich quadrant is no longer useful, delaying the complete attentional reprioritization of the new-rich quadrant.

The instruction group, as well as the no-instruction group, showed persistent prior bias until the switching phase, despite possessing explicit knowledge that a target now does not appear often at the prior rich quadrant but it appears at every quadrants with the same probability. This is because the instruction group, despite relying heavily on explicit knowledge, also obtained habit-like attentional bias by repetitively finding a target in the rich quadrant. Explicit knowledge on target probability does not fully override implicit learning (Jiang et al., 2014), enabling experience-driven attentional learning. The critical difference between the instruction and no-instruction group should be focused on the novel attentional bias than a prior bias. While the instruction group showed greater attentional bias to the new-rich quadrant using explicit knowledge, the no-instruction group showed a similar extent of attentional bias to the new-rich and old-rich quadrants. The attentional control resources the participants rely on seem to modulate the sensitivity or the learning speed of the shifted regularity.

In some cases, we can obtain sudden explicit knowledge that conflicts with a previously implicit one. An implicitly obtained attentional bias is no longer helpful then, and attentional bias should be explicitly directed to another location. In this case, implicit and habitual attentional bias toward the target’s previous location is expected to largely influence novel explicit attentional bias. Habitual attentional bias is less susceptible to outcome devaluation (Jiang & Sisk, 2019; Jiang et al., 2013b) and is not discarded despite a sudden instruction of a novel regularity (Jiang et al., 2014). While maintaining a prior attentional bias, a new bias would emerge to a novel rich quadrant, using top-down knowledge of the target’s location. This expected result would imply that goal-driven attention and habit-like attention are dissociable.

The current study addressed the three major concerns that Vadillo et al. (2020) and Giménez-Fernández et al. (2020, 2021) raised regarding LPL studies. First, LPL by habit-like attention was found to be relatively inflexible compared with goal-driven attention. Even when the learned bias was no longer a suitable search strategy due to a changed statistical environment, the existing bias was not rapidly discarded. Second, LPL by habit-like attention is implicit, and its biasing pattern is unlike search by explicit goal-driven attention. While goal-driven attention showed the flexibility of attentional prioritization, habit-like attention showed inflexibility. Third, the different attentional biasing according to awareness state was evident and apparent even with a 1/5 sample size from Giménez-Fernández et al. (2020), so long as the sample size was large enough to provide sufficient statistical power.

This study is limited to observe a definite inflexibility of habit-like attention, which is not expected. The current study tested attentional updating within two blocks of the switching phase. Longer switching blocks would result in more attentional prioritization of the no-instruction group to the new-rich quadrant than the old-rich quadrant, as was in the instruction group (Jiang et al., 2013b). Accordingly, the flexibility and inflexibility of habit-like attention and goal-driven attention should be understood to a relative extent, and not as categorical concepts. The terms flexibility and inflexibility in this study are equivalent to relative responsiveness to the spatiotemporal context. As habit-like attention was updated more slowly, it is more inflexible; as goal-driven attention was updated more rapidly, it is more flexible.

Converging evidence supports the claim that goal-driven and habit-like attention are based on distinctive attentional mechanisms (e.g., Addleman et al., 2018). This study is accordant to this stream because different biasing strategies were used depending on the awareness state of target regularity. While goal-driven attention quickly updated attentional bias and reduced previous bias, habit-like attention showed an additive biasing strategy that addressed a similar extent of attentional priority to the old- and new-rich quadrants. While goal-driven attention is relatively flexible to regularity variance, habit-like attention, once learned, is relatively inflexible to regularity change. The results of this study are consistent with the existing model of Jiang (2018), which dissociates habit-like attention and goal-driven attention in its temporal and spatial (in)flexibility.

The need for a revised taxonomy of attentional resources is not new. Rather than top-down and salience-driven attention, other factors such as structure, value (Anderson, 2013), selection history (Awh et al., 2012), and scene (Wolfe et al., 2011) can guide spatial attention (Wolfe & Horowitz, 2017). While the number of attentional shifts enabled LPL, the source of statistical knowledge also altered the attentional prioritization structure. The third category of attentional resources also plays a role in spatial attention, but its specific mechanism should be investigated further.

The current study possesses practical implications of LPL in various fields. Explicit or implicit training seems to be used appropriately in critical situations. The habitual LPL paradigm can be used to develop novel rehabilitation or educational programs to complement attentional orienting of those with impaired goal-driven attention. In other cases where a search relies on a rapid readjustment of spatial bias, explicit knowledge that guides spatial attention would be helpful. In a dynamic search circumstances like web surfing, playing games, driving, searching for a criminal and so on, explicit knowledge about the target’s location would facilitate attentional reorienting.

## Conclusion

The current study investigated the spatiotemporal inflexibility of LPL by habit-like attention compared with flexible bias updating by goal-driven attention. The results demonstrate that goal-driven and habit-like attention should be distinguished. Goal-driven attention relatively relies on explicit and flexible processes, while habit-like attention relatively relies on implicit and inflexible processes. Both goal-driven and habit-like attention can affect visual search strategies, but in different ways. As a possible third category of the attentional resource, the distinguishing characteristics of habit-like attention should be further investigated.

## Supplementary Information

**Additional file 1.** Supplementary Results.

## Data Availability

The datasets generated and analyzed during the current study are available in the Open Science Framework repository, https://osf.io/kez9r/?view_only=08489e28819143248ea1ae3ea62fe78c. Experiment and data analysis R codes generated during the current study are available from the authors on reasonable request.

## Abbreviations

LPL: Location probability learning
RT: Reaction time
ms: Milliseconds
s: Seconds
4AFC task: Four-alternative forced choice task
GLMM: Generalized linear mixed-effect model
