# Supplementary material for: Habit-like attentional bias is unlike goal-driven attentional bias against spatial updating
Source: Cogn Res Princ Implic. 2022 Jun 17;7:50. doi: 10.1186/s41235-022-00404-7 (PMC9206057; doi:10.1186/s41235-022-00404-7)
Supplement: Supplementary file 1 — Additional file 1. Supplementary Results. [file 41235_2022_404_MOESM1_ESM.doc]

**Supplementary Results**

**Repetition Priming Effects in LPL**

Most location probability learning studies have previously included additional search blocks with equal target frequency to segregate persistent learning effect from a short-term repetition priming effect (Brascamp et al., 2011; Maljkovic & Nakayama, 1996). The switching phase of the current study involves the target’s frequent presence in the new-rich quadrant due to the regularity change. Consequently, it is hard to discriminate the long-term attentional bias from the short-term repetition priming effect. Therefore, additional analysis was conducted on the switching phase by removing the *n*th trials, wherein target quadrant was identical to the *n*-1th trials. Removing repeated trials numerically increased the overall RTs, and the descriptive statistics for mean and SD are reported in Table S1.

Table S1. Descriptive statistics of RTs in milliseconds of each phase and target’s location by awareness state group and trial types.

| Group | Training Phase | | Switching Phase | | |
| --- | --- | --- | --- | --- | --- |
|  | Rich | Sparse | Old-Rich | New-Rich | Both-Sparse |
|  | all trials (*N* = 32) | | | | |
| Instruction | 924.45 (351.7) | 1153.39 (367.77) | 1045.59 (292.98) | 847.54 (301.11) | 1114.02 (213.29) |
| No-Instruction | 820.56 (78.67) | 987.88 (152.33) | 846.16 (128.14) | 812.25 (164.15) | 965.94 (186.26) |
|  | all trials (*N* = 60) | | | | |
| Instruction | 877.62 (291.81) | 1136.34 (305.51) | 983.59 (262.19) | 851.77 (270.84) | 1081.34 (211.55) |
| No-Instruction | 808.97 (92.63) | 1002.06 (150.3) | 844.17 (123.28) | 832.76 (140.01) | 968.71 (179.37) |
|  | location-repeated trials excluded (*N* = 32) | | | | |
| Instruction | 942.39 (340.25) | 1163.56 (358.81) | 1030.28 (245.32) | 859.05 (287.61) | 1138.99 (231.92) |
| No-Instruction | 845.7 (92.93) | 1002.02 (162.17) | 853.71 (133.35) | 852.81 (198.67) | 969.83 (202.19) |
|  | location-repeated trials excluded (*N* = 60) | | | | |
| Instruction | 894.75 (291.77) | 1147.89 (297.86) | 975.86 (235.59) | 867.6 (271.38) | 1100.3 (223.24) |
| No-Instruction | 830.61 (105.59) | 1007.88 (151.88) | 851.86 (138.38) | 868.66 (160.03) | 968.72 (185.63) |

*Note*. The means and standard deviations in parenthesis of RTs.

First, we analyzed the RTs with 32 valid data sets considering the responses on the awareness stage’s questionnaire. The fixed effect of the target’s location was significant (*χ^2^*(2) = 128.927, *p* < .001), however, the interaction between the target’s location and the awareness state was statistically different, *χ^2^*(2) = 20.286, *p* < .001. Overall RTs by awareness state were not statistically different, *χ^2^*(1) = 1.959, *p* = .162. Specifically, the instruction group showed faster target detection in the new-rich quadrants compared with the old-rich, *z* = -6.164, *p* < .001, and the both-sparse quadrants, *z* = 10.880, *p* < .001. The old-rich and new-rich conditions also showed significantly different RTs, *z* = 2.875, *p* = .012. Though RTs in the old-rich quadrant were faster than the both-sparse quadrants, the new-rich quadrant gained more attentional priority than the old-rich quadrant. For the no-instruction group, RTs of the new-rich condition were faster than the both-sparse conditions, *z* = 5.413, *p* < .001. RTs of the old-rich condition were faster than the both-sparse condition, *z* = 4.690, *p* < .001, but were not statistically different from the new-rich condition, *z* = .257, *p* < 1.

Second, we analyzed 60 full data sets regardless of the responses at the awareness stage. Trials after excluding location repetitions yielded similar results to the original analysis. While RTs by awareness state were not significantly different, *χ^2^*(1) = 1.642, *p* = .200, the fixed effect of the target’s location was significant, *χ^2^*(2) = 191.048, *p* < .001. Target’s location × awareness state interaction was also significant, *χ^2^*(2) = 24.255, *p* < .001. A post-hoc analysis again demonstrated the fastest detection times in the new-rich condition than the old-rich, *z* = -5.199, *p* < .001, and the both-sparse conditions, *z* = 11.987, *p* < .001 for the instruction group. The old-rich quadrant also demonstrated faster RTs than the both-sparse quadrants, *z* = 4.694, *p* < .001. In contrast, the no-instruction group showed a similar extent of attentional bias toward the old-rich and new-rich quadrants, *z* = 1.286, *p* = .595. This bias was evidenced by the faster RTs than the both-sparse conditions, old-rich *z* = 6.944, *p* = .001; new-rich *z* = 6.881, *p* < .001. The results are demonstrated in Figure S1.

**Figure S1**

*Mean RTs of the instruction and no-instruction group excluding the quadrant-repeated trials*


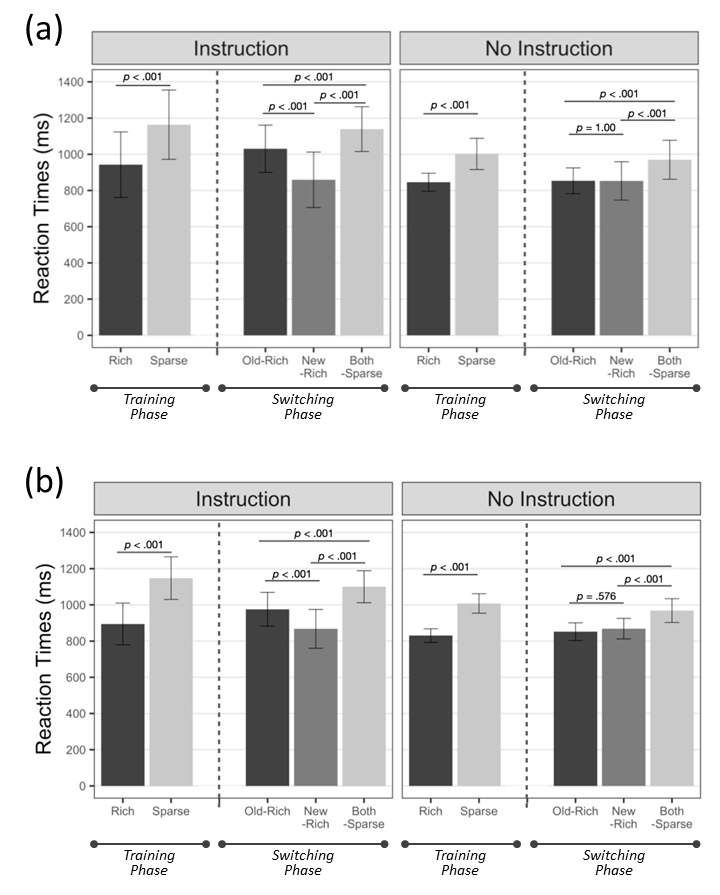


*Note*. (a) RTs of data sets whose awareness score was reflected (*N* = 32). (b) RTs of the full data sets whose awareness score was not reflected (*N* = 60). Error bars represent 95% confidence intervals.

**Analysis on accuracy and RTs trade-off**

As reported in the article, the mean accuracy was 98.71% (*SD* = 1.28%) for the instruction group and 96.62% (*SD* = 3.50%) for the no-instruction group (for 32 data sets). Even though search displays were provided until the response was made and the response was obvious, we found a significant difference in the accuracy level by the awareness state groups, *t*(30) = -.240, *p* = .032. Differences on accuracy for the groups were also found when all 60 data sets were all considered, instruction group *M* = 98.65%, *SD* = 1.18%; no-instruction group *M* = 96.87%, *SD* = 3.19%, *t*(58) = -2.842, *p* = .008.

The difference in accuracy levels by groups can cause RTs-accuracies trade-off, reducing the reliability of RTs analysis. Thus, the spatial updating of instruction and no-instruction groups was tested after complementing this possible trade-off by dividing RTs by the accuracy level of individual participants. This novel index represents the general search performance.

An accuracy and RT value were calculated by individual participants, and a novel index was created by dividing RTs by accuracy. This value was submitted to GLMM as a dependent variable, and the target’s location and awareness state were submitted as fixed effects.

In the training phase, when the awareness score was considered (*N* = 32), RT / accuracy value was not different by groups, *χ*^2^(1) = .323, *p* = .570. The target’s location factor was significant, *χ*^2^(1) = 58.637, *p* < .001. The interaction between the target’s location and awareness state was not significant, *χ*^2^(1) = .031, *p* = .859.

In the switching phase, the value by awareness state was also not significant, *χ*^2^(1) = .936, p = .333. The target’s location factor was significant, *χ*^2^(2) = 38.894, *p* < .001, and the interaction between the target’s location and awareness state was also significant, *χ*^2^(2) = 4.746, *p* = .093.

Though the significance of the interaction was at a marginal level, a post-hoc analysis was conducted to find out if this additional analysis bears a similar trend of RT/accuracy compared with the original RT. For the instruction group, the new-rich’s value was significantly lower than the old-rich’s, *z* = -4.627, *p* <.001. Old-Rich’s value was not significantly different from both-sparse’s value, *z* = .939, *p* < .1. New-rich’s and both-sparse’s values were significantly different, *z* = 5.527, *p* < .001. For the no-instruction group, the old-rich’s and new-rich’s values were not significantly different, *z* = -1.364, *p* = .518. Both-sparse’s value were significantly higher than old-rich’s value, *z* = 3.008, *p* = .008, and new-rich’s value, *z* = 4.347, *p* < .001.

When full data sets were included in the analysis, we obtained a similar trend of results. For the training phase, the fixed effect of the awareness state groups was not significant, *χ*^2^(1) = .413, *p* = .520. The fixed effect of the target’s location was significant, *χ*^2^(1) = 133.380, *p* < .001. The fixed effect of the interaction between the target’s location and awareness state was not significant, *χ*^2^(1) = .148, *p* = .701.

For the switching phase, the fixed effect of the awareness state was not significant, *χ*^2^(1) = .852, p = .356. The fixed effect of the target’s location was significant, *χ*^2^(2) = 59.036, *p* < .001. The interaction between the target’s location and awareness state was marginally significant, *χ*^2^(1) = 5.542, *p* = .063.

A post-hoc analysis also revealed a similar trend of statistical tests. For the instruction group, the new-rich value was significantly lower than the old-rich value, *z* = -4.627, *p* < .001. Old-Rich and both-sparse values were not different, *z* = .939, *p* < .1. New-Rich and both-sparse values were significantly different, *z* = 5.527, *p* < .001. For the no-instruction group, unlike the instruction group, the old-rich and new-rich values were not significantly different, *z* = -1.364, *p* = .518. Both-sparse’s value was statistically different from old-rich’s, *z* = 3.008, *p* = .008, and new-rich, *z* = 4.347, *p* < .001.

Overall, a similar trend of the statistical test was found when accuracy level was considered in RT analysis. While the instruction group showed a generally better performance at the new-rich quadrant than old-rich quadrant, the no-instruction group showed similar search performance at the old-rich and new-rich quadrants.

**References**

Brascamp, J. W., Pels, E., & Kristjánsson, Á. (2011). Priming of pop-out on multiple time scales during visual search. *Vision Research*, *51*(17), 1972–1978. https://doi.org/10.1016/j.visres.2011.07.007

Maljkovic, V., & Nakayama, K. (1996). Priming of pop-out: II. The role of position. *Perception & Psychophysics*, *58*(7), 977–991. https://doi.org/10.3758/BF03206826
